# The Use of CT-Guided Marking for the Laparoscopic Resection of a Solitary Retroperitoneal Metastasis of Colon Cancer

**DOI:** 10.1089/cren.2018.0049

**Published:** 2018-08-01

**Authors:** Hideto Ueki, Takuya Fujimoto, Masato Okuno, Yuji Kusuda, Isao Taguchi, Yasushi Itou, Sawami Kiyonaka, Gaku Kawabata

**Affiliations:** ^1^ Division of Urology, Kansai Rosai Hospital, Amagasaki, Japan.; ^2^ Division of Radiology, Kansai Rosai Hospital, Amagasaki, Japan.; ^3^ Division of Anesthesia, Kansai Rosai Hospital, Amagasaki, Japan.

**Keywords:** solitary metastasis to the retroperitoneum, small tumor, CT-guided marking, laparoscopic resection of tumor

## Abstract

***Background:*** CT-guided marking technique is rarely used in abdominal or urologic surgery. We developed and performed a marking technique for a small tumor, undetectable by ultrasound, using CT guidance before laparoscopic resection of the tumor.

***Case Presentation:*** A 73-year-old woman with a history of breast cancer underwent right colectomy with D3 lymph node dissection for ascending colon cancer. Five years after the operation, a solitary tumor was found in the right pararenal region of the retroperitoneal space on enhanced abdominal CT. The tumor was 20 mm in diameter and undetectable by ultrasound, so we performed a marking technique using CT guidance before the operation. Placing the patient in a prone position on the CT table, a 22-gauge needle was inserted into the Gerota's fascia percutaneously and a mixed fluid containing India ink and Iopamidol was injected para to the tumor by the radiologist. During the surgery, the marker was clearly identified and the cutting line was determined to ensure a sufficient surgical margin. The tumor was laparoscopically resected as planned. The histopathologic diagnosis was adenocarcinoma, compatible with metastasis of colon cancer. The postoperative course was uneventful and the patient remained free of disease at 10 months after surgery.

***Conclusion:*** When resecting small tumors or tumors with an irregular margin, a marking technique is conducted before the surgery. But, preoperative CT-guided marking has not been applied generally for resection of intraabdominal lesion yet. CT-guided marking can be effective when performing minimally invasive and curable surgery on small tumors. This is the first report of an effective CT-guided marking before retroperitoneal laparoscopic tumorectomy. We believe that this technique provides an important therapeutic option for small tumors that may be undetectable by ultrasound.

## Introduction and Background

The liver and lung is a common site of colon cancer metastasis, the frequency being 10.7% and 1.6%, respectively. However, solitary metastasis to the retroperitoneum is rare.

In general, metastasis to the retroperitoneum is the last scene of invasive cancers, and a metastatic retroperitoneal tumor that can be resected is evermore rare. Because of that, effective treatment, including postoperative adjuvant chemotherapy, has yet to be established. We report a rare case of solitary metastasis to the retroperitoneum from colon cancer resected 5 years after the primary operation.

When resecting small tumors or tumors with an irregular margin, a marking technique is conducted before the surgery. CT-guided marking techniques are common in pulmonary surgery, but it is rarely used in abdominal or urologic surgery.^1^ We developed and performed a marking technique for a small tumor, undetectable by ultrasound, using CT guidance before laparoscopic resection of the tumor.

## Presentation of Case

A 73-year-old woman with a history of breast cancer underwent right colectomy with D3 lymph node dissection for ascending colon cancer. The final finding was adenocarcinoma, tub1>tub2, pSE, int, INFb, ly1, v1, pPM0, pDM0, pRM0, pT4N1M0, pStageIIIa, pR0, and CurA. Postoperative adjuvant chemotherapy consisting of FOLFOX and Capecitabine was performed for 6 months.

**FIG. 1.**
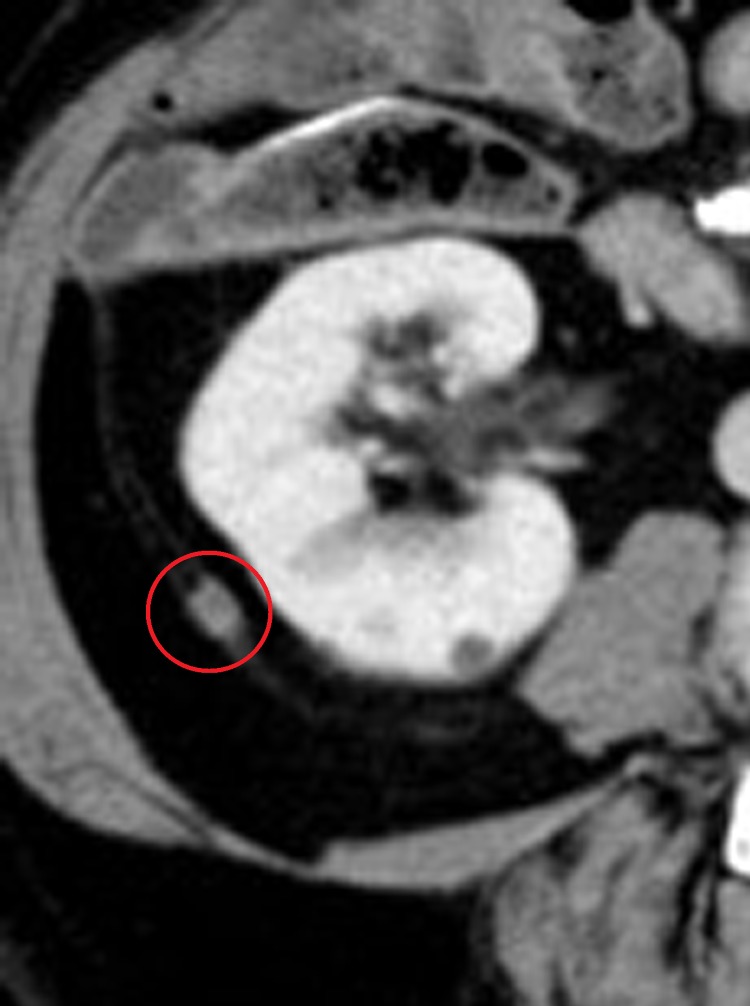
CT showed a solitary tumor in the right pararenal region of the retroperitoneal space 5 years after the operation (*red circle*).

Five years after the operation, a solitary tumor was found in the right pararenal region of the retroperitoneal space on enhanced abdominal CT scan (Fig. 1). In retrospect, this tumor was already present 15 months ago and had been growing slowly but steadily. There was no increase in the serum level of carcinoembryonic antigen. Fluorodeoxyglucose (FDG) positron emission tomography CT showed lesion accumulation of 18F-FDG, but it could not be detected by ultrasound.

**FIG. 2.**
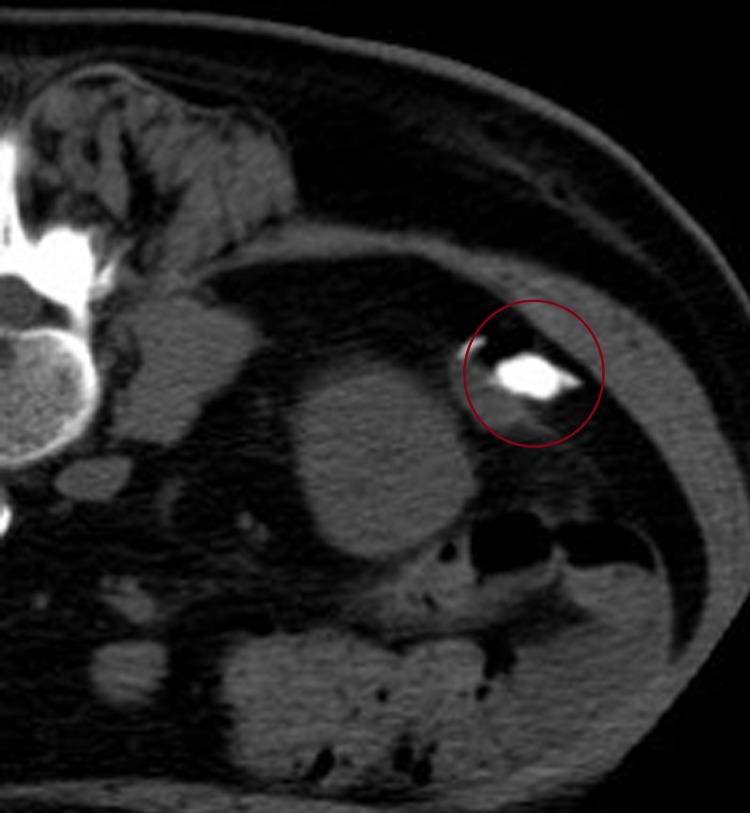
Placing the patient in a prone position, a 22-gauge needle was inserted into the Gerota's fascia percutaneously and India ink and iopamiron were injected close to the tumor. The *red circle* shows Iopamidol which was injected para to the tumor.

**FIG. 3.**
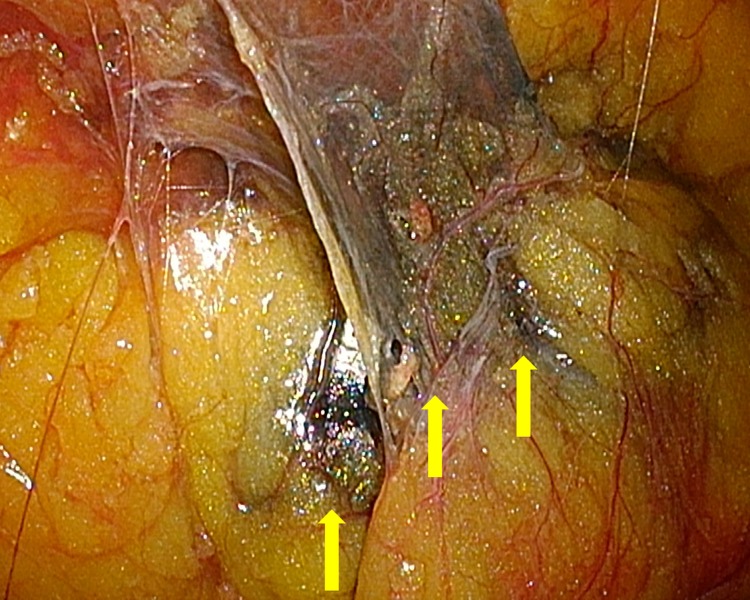
Intraoperative findings. India ink which stained perirenal fat could be confirmed macroscopically. *Yellow arrows* show the edge of the marking of India ink on flank pad.

Considering it has diagnostic and therapeutic significance to resect the tumor, under the diagnosis of a solitary metastatic tumor from the colon cancer, we decided to perform a retroperitoneal laparoscopic resection of the tumor. To resect the tumor, we planned to mark it using CT guidance before surgery. Placing the patient in a prone position on the CT table, a 22-gauge needle was inserted into the Gerota's fascia percutaneously and 0.7 mL of a mixed fluid containing India ink and Iopamidol (Iopamiron^®^; Bayer Yakuhin) (5:1) was injected para to the tumor by the radiologist (Fig. 2). Because the surgery was scheduled on Monday, we performed retroperitoneal laparoscopic resection 3 days after the marking. Since India ink remains in place for several days, it was easy to recognize it during surgery without fluoroscopy (Fig. 3).

**FIG. 4.**
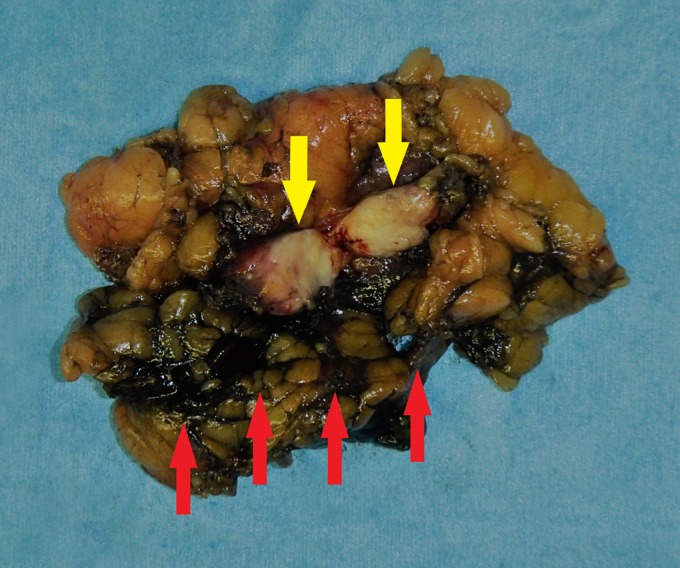
The resected tumor was a light yellow solid nodule (*yellow arrows*). An appropriate surgical margin was obtained in the resected specimen. The India ink marking on the surface of tumor was easily visualized (*red arrows*).

After identification of the India ink marker, the cutting line was determined on the Gerota's fascia surface to ensure a sufficient surgical margin. We started an incision from the flank pad around the marking and exfoliated the tumor with the surrounding fat, identifying the kidney capsule. Gross examination showed a light yellow solid nodule measuring 20 mm (Fig. 4). Histologic examination showed gland formation consisting predominantly of atypical columnar epithelial cells. It was similar to the previous pathologic findings from the colon cancer. Immunohistochemical analysis revealed that the neoplastic cells were positive for Cytokeratin 20 (CK20) and negative for Cytokeratin 7 (CK7). The histopathologic diagnosis was adenocarcinoma, compatible with metastasis of colon cancer. The postoperative course was uneventful and the patient remained free of disease at 10 months after surgery.

It is well known that the most common metastatic site of colon cancer is the liver, followed by the lung. There are few reports describing metastasis to the retroperitoneum and solitary ones are more rare. There have been only 11 cases of solitary metastatic retroperitoneal tumor reported in Japan, and 5 cases were from colon cancer.

There are several metastatic pathways, including dissemination, hematogenous, and lymphoid metastasis. In this case, the metastatic tumor was observed solitarily within the adipose tissue in the Gerota's fascia, so the probability of dissemination or hematogenous metastasis is low. Histologic examination of the ascending colon cancer showed lymph node metastasis (pT4N1M0), but the location of the metastatic tumor was displaced from the lymphatic flow. Although it is difficult to elucidate the mechanism of metastasis, lymph node invasion (ly1) and vein invasion (VB1) are an unlikely mode of metastasis in our case. At any rate, since the prognosis of retroperitoneal metastatic tumor depends on the presence of metastasis of other sites, it was considered that there was indication for surgical treatment if it was solitary metastasis.

CT-guided marking with the hookwire has been applied for video-assisted thoracoscopic surgery in patients with a small pulmonary tumor, which is hard to identify intraoperatively.^2^ In urology, it is said that preoperative CT- or MRI-guided marking is helpful for decreasing complications of tumor localization for renal cell carcinoma. However, preoperative CT-guided marking has not been applied generally for resection of intraabdominal lesion yet. Regarding size, Ishimaru et al. reported the usefulness of this method in a 15 mm solitary retroperitoneal metastasis of pediatric pancreatoblastoma,^3^ and it is said that a small pulmonary tumor less than 10 mm is good indication. Preoperative CT-guided marking for pulmonary nodule is common, however, only 46 reports have been described in literature, the site being 40 lungs, 3 breasts, 1 liver, 1 spine, and 1 subcutaneous. The tumor diameter was 1.5–2.0 cm, and the marking techniques used were lipiodol or indocyanine green (ICG) marking, wire marking, and combinations of wire and lipiodol marking. Lipiodol is a lipid-soluble contrast material that is used for transcatheter arterial chemoembolization for hepatocellular carcinoma and lymphangiography. In our case, radiologists in our hospital were familiar with using India inks, and so we chose to use it. As India ink is nonabsorbable, adverse events such as focal peritonitis and adhesion ileus caused by intraperitoneal leak may be a problem. On the contrary, although ICG is absorptive, there is no problem in dyeability, and it is reported to be effective if the period from marking to surgery is within 8 days. We think that it was possible to use ICG even in this case. The limitation of this method is that the dyeing solution may become extensive and obscure, and cooperation of the radiology department is necessary. In addition, it may cause infection and bleeding before surgery. In our case, guiding marker placement was performed without any complications and was useful for detection of the target.

Ishimaru et al. have already reported preoperative CT-guided marking for retroperitoneal metastasis.^3^ They used a mixture of ICG, ethiodized oil, and lidocaine hydrochloride jelly, which reportedly remained in place and was useful for intraoperative identification of pulmonary nodules 60–80 min after injection.

Their operation was different from our case in that it was laparotomy and pediatric case.

Laparoscopic surgery is easier to identify the dye than laparotomy, however, the presence or absence of tactile sensation is big difference. Although Monden et al. reported two cases of partial hepatectomy using hookwire marking under CT guidance,^4^ our case is the first report to describe the usefulness of a CT-guided marking in laparoscopic resection of tumor for solitary retroperitoneal metastasis.

In conclusion, this technique can be effective when performing minimally invasive and curable surgery on small tumors. We think it is good indications for a tumor buried in fat and less than 3 cm in diameter which could not be detected by ultrasound. Taking the risk of bleeding into consideration, it is important that the tumor is far from the critical organs or great vessels.

Currently the number of reported cases is small, but if the procedure can be adapted to other cases, there is a possibility that more patients will benefit in the future.

## Conclusion

CT-guided marking can be effective when performing minimally invasive and curable surgery on small tumors.

## Abbreviations Used

CT: computed tomography
FDG: fluorodeoxyglucose
ICG: indocyanine green
MRI: magnetic resonance imaging
